# Enhanced Electron Scattering upon Ion Relocation in BaVS_3_ at 69 K

**DOI:** 10.3390/e21080813

**Published:** 2019-08-20

**Authors:** Ferenc Márkus, Bence G. Márkus

**Affiliations:** 1 Department of Physics, Budapest University of Technology and Economics, P.O. Box 91, H-1521 Budapest, Hungary; 2 Department of Physics, Budapest University of Technology and Economics and MTA-BME Lendület Spintronics Research Group (PROSPIN), P.O. Box 91, H-1521 Budapest, Hungary

**Keywords:** BaVS_3_, metal-insulator transition, enhanced electron scattering

## Abstract

The present study deals with the anomalous heat capacity peak and thermal conductivity of BaVS${}_{3}$ near the metal-insulator transition present at 69 K. The transition is related to a structural transition from an orthorhombic to monoclinic phase. Heat capacity measurements at this temperature exhibit a significant and relatively broad peak, which is also sample dependent. The present study calculates the entropy increase during the structural transition and we show that the additional entropy is caused by enhanced electron scattering as a result of the structural reorientation of the nuclei. Within the model it is possible to explain quantitatively the observed peak alike structure in the heat capacity and in heat conductivity.

## 1. Introduction

**Figure A1:**
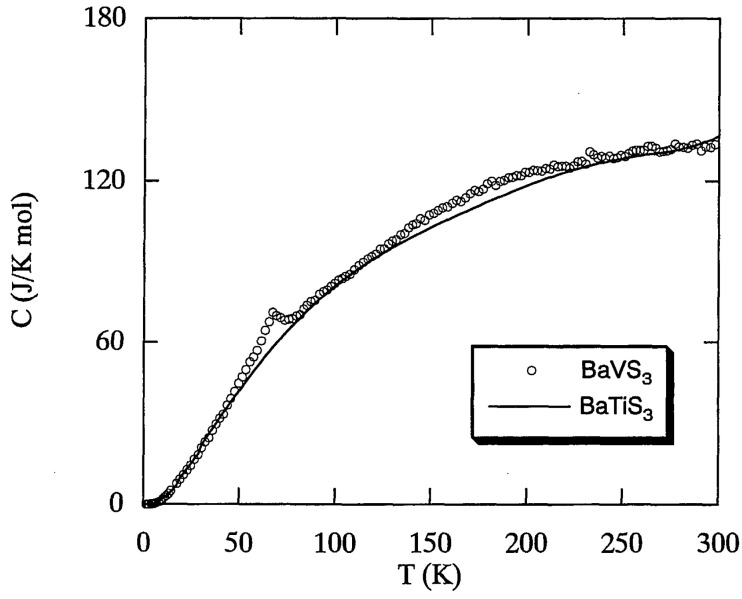
Specific heat of BaVS${}_{3}$ (open circles) and BaTiS${}_{3}$ (solid curve, nonmagnetic reference). Adopted from Reference [2] (c) (1996) The Physical Society of Japan.

**Figure A2:**
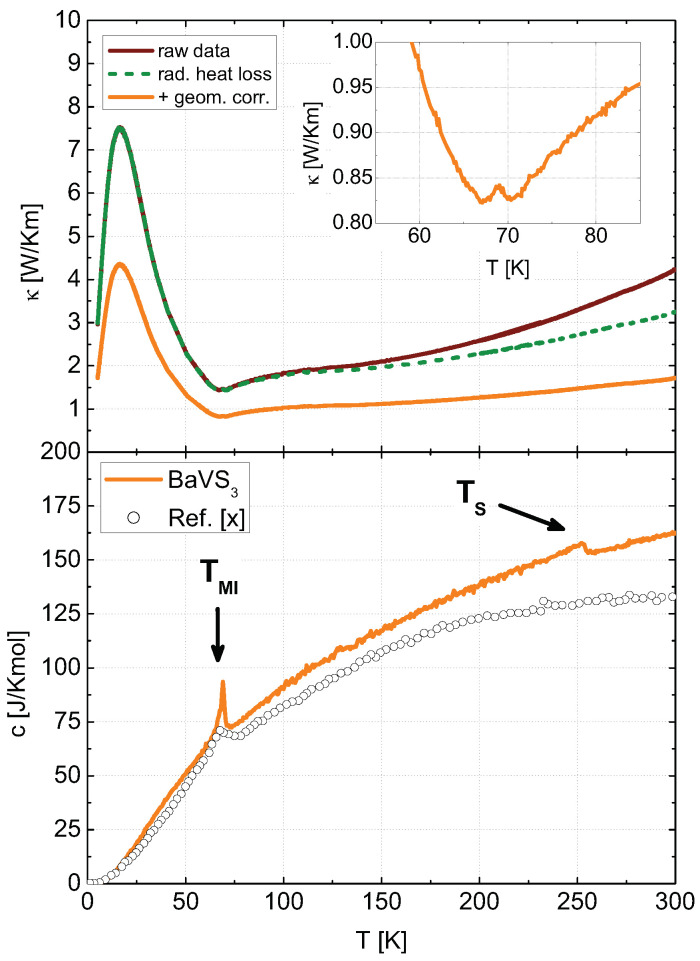
Upper panel: thermal conductivity of BaVS${}_{3}$. Data without/with considering the effect of radiative heat loss (upper solid line/dashed line) and final data when the geometric correction has also been taken into account (lower solid line). Inset: anomaly in the close vicinity of the phase transition. Lower panel: specific heat of BaVS${}_{3}$. Open circles represent former results adopted from Imai et al. [2] Besides the insulator to metal transition, the present study also reveals a contribution associated with the structural transition at $T_{S}$. Adopted from Reference [6].

Materials that bear the ABX${}_{3}$ structure are continuously studied due to their novel properties, which have potential applications, for example MOSFET (metal-oxide-semiconductor field-effect transistor) device fabrication [1]. One member of this family is barium vanadium sulfide (BaVS${}_{3}$), which has unique electronic properties such as metal-insulator transition [2,3,4,5,6], “bad metal” behavior [7], magnetic field induced structural transition [8], charge density waves (CDW) [5] and paramagnetic-antiferromagnetic phase transition [9]. It is experimentally [2,3,4,5,6] and theoretically [10,11] verified that the barium vanadium sulfide (BaVS${}_{3}$) has an orthorhombic to monoclinic structural transition during the metal-insulator (MI) phase transition at a temperature of approximately $T_{\mathrm{MI}}=69$ K. The given structural transition relates to an extensive regime of one-dimensional lattice fluctuations. The timescale of the phase transition is probably in the same order, a couple nanoseconds, as in a CDW transition, which is indeed involves a reorientation and is 1 ns in the present material as described in Reference [5]. Detailed measurements [2,6] are elaborated to determine the changes in the measurable physical quantities, like thermal conductivity and specific heat. Since the specific heat is a sensitive indicator of phase transitions it is worth focusing on its behavior. A significant but relatively broad peak appears at $T_{\mathrm{MI}}$ in the specific heat measurements, as can be seen in Figures 2 and 4 of Reference [2] and Reference [6], respectively. For clarity these figures are presented in the Appendix A as Figure A1 and Figure A2. This peak is absent in general in MI transitions. The ordered magnetic–paramagnetic phase transition [12,13], the charge density waves near the Peierls transition [14,15], charge density and spin wave [16,17] are also good candidates in the explanation; however, the caused effect of the previously mentioned phenomena is too small, even when their effects are summed up. Until now, no adequate explanation exists for this behavior. The width of the peak may have sample quality (e.g., S-component ratio [18]) and sample size dependence.

## 2. The Entropy Increase in the Metal-Insulator Transition

**Figure A3:**
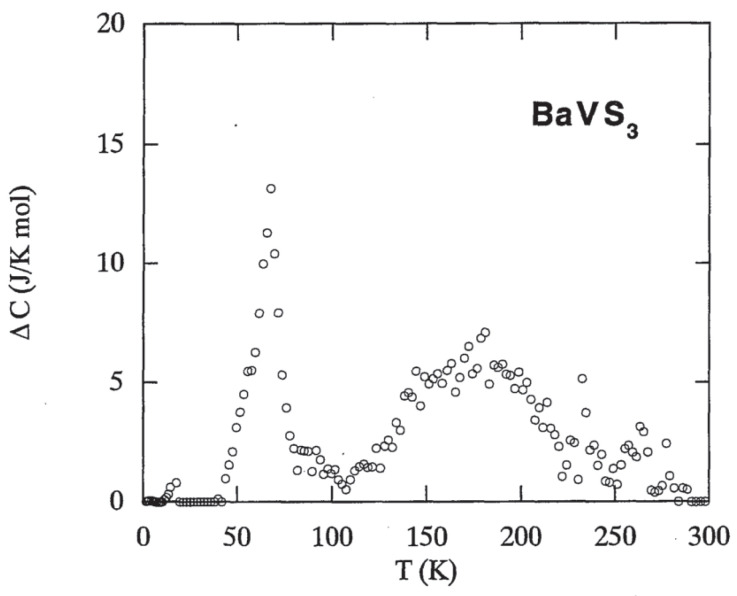
Extra specific heat of BaVS${}_{3}$, estimated by subtracting the specific heat of BaTiS${}_{3}$ from the specific heat of BaVS${}_{3}$. Adopted from Reference [2] (c) (1996) The Physical Society of Japan.

**Figure A4:**
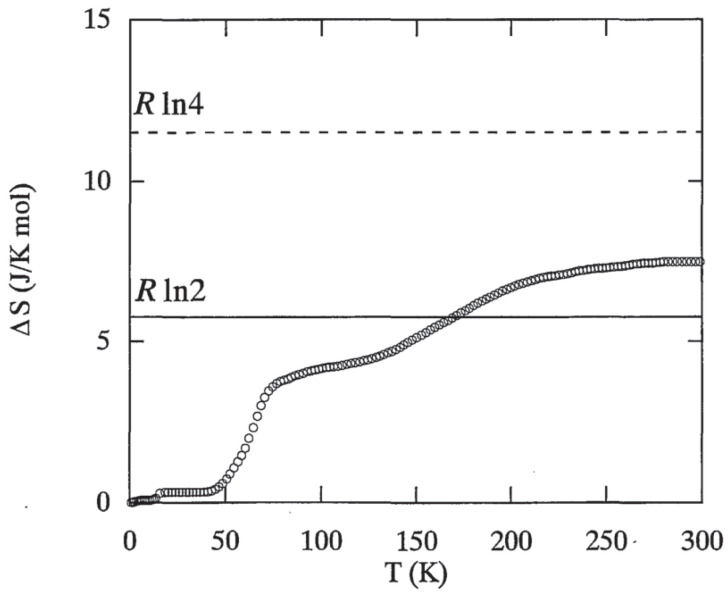
Extra entropy ΔS of BaVS${}_{3}$ as a function of *T*. Adopted from Reference [2] (c) (1996) The Physical Society of Japan.

According to self-consistent electronic structure calculations [19] the specific heat of the metallic regime of BaVS${}_{3}$ agrees well with that of the insulating BaTiS${}_{3}$. The difference of the specific heat among the samples can be calculated, as shown in Figure 3 of Reference [2], also depicted in Figure A3. Furthermore, from the obtained curve the extra entropy can be also extracted, presented in Figure 4 of Reference [2], as well as in Figure A4. From the resulted plot the authors claim that, the steepness of the curve is the highest at around the MI transition point, $T_{\mathrm{MI}}=69$ K and a 4.7 J/K mol extra entropy difference is also extracted from the curve in the range of 40–100 K.

Later on, the authors conclude that this entropy increase is rather close to the value Rln(2S+1)=5.76 J/K mol with S=1/2, which would mean that the degree of freedom for S=1/2 spins has a contribution just above the metal-insulator transition. However, the difference between the experiment and the expected increment from a spin half excitation is more than 22%, which is rather large compared to the error of the measurements. Furthermore, the excess entropy in the case of the other sample [6] is two times higher, than the one observed before and it is clear that this can not be explained by the upper mentioned argument.

**Figure 1:**
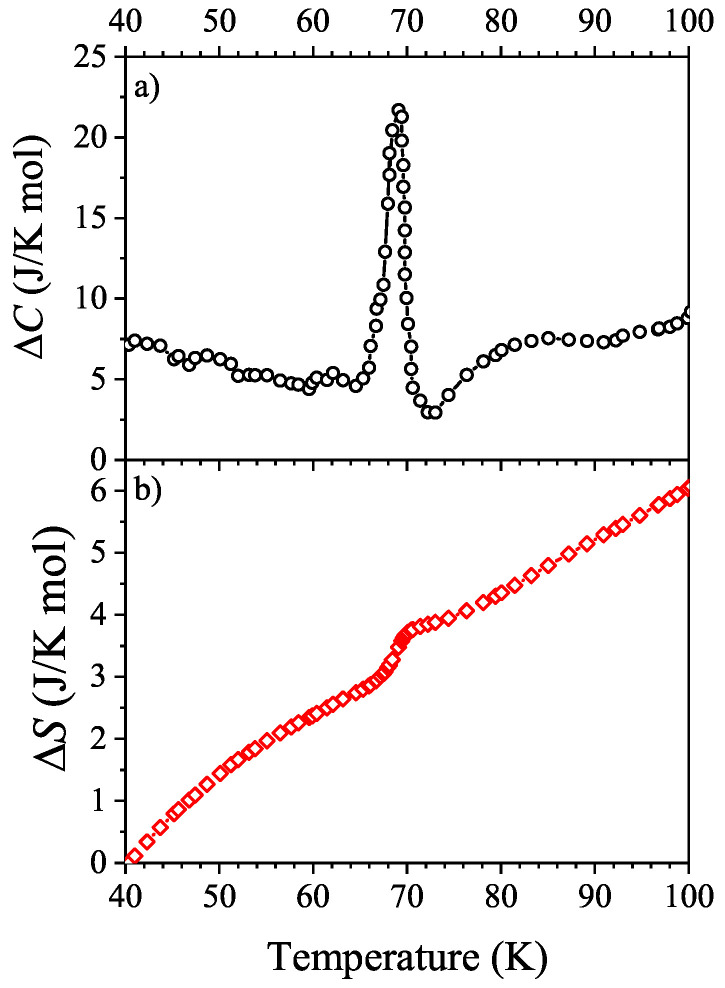
(**a**) The difference of specific heat near the metal-insulator transition between the two BaVS${}_{3}$ samples presented in Reference [6] and in Reference [2]. (**b**) Extra entropy in the temperature range of 40–100 K extracted from the specific heat difference.

Comparing the plots of specific heat in Figure 4 of Demkó et al. [6] (Figure A2 of the Appendix A), it can be seen clearly that the peak observed in the heat capacity is more dominant, pronounced but also sharper, narrower than the one present in the previous article [2]. Extracting the empirical data from the plot and adopting the idea presented in Reference [2], the difference of specific heat among the two measurements on BaVS${}_{3}$ is plotted in Figure 1a in the relevant temperature range of 40–100 K. In Figure 1b the explicit entropy excess between the two samples is calculated.

The additional extra entropy is 6.0 J/K mol between the two stoichiometrically identical BaVS${}_{3}$ samples in the range of 40 to 100 K. Compared to the reference BaTiS${}_{3}$, one sample has 4.7 J/K mol excess entropy at 100 K [2] while the other presents an excess of 10.7 J/K mol [6]. This significant difference suggests that the reason for extra entropy must be an additional or a different process.

## 3. Contribution of Internal Electron Scattering to the Specific Heat Peak

The finite width of the peak may suggest oscillations and scattering of the electrons around the ions during the structural transition [2], which might be related to the dynamical behavior of the process. However, the damped oscillation of the ion cores, for example, the acoustic phonon modes can fairly contribute to the internal energy. On the other hand, when electron scattering is taken into account, the core rearrangement can induce an enhancement in the electron scattering rate. We suggest that the effect arises because of the structural transition and the dramatic change of the band-structure. The mechanism presented here takes a model where a free electron gas is present—as approaching from the metallic phase—with the added oscillations arising from core relocation upon phase transition. During the process, the internal energy and the specific heat is calculated. The quantitative model reflects well that these oscillations may produce such a temperature dependence of specific heat as it is measured.

**Figure 2:**
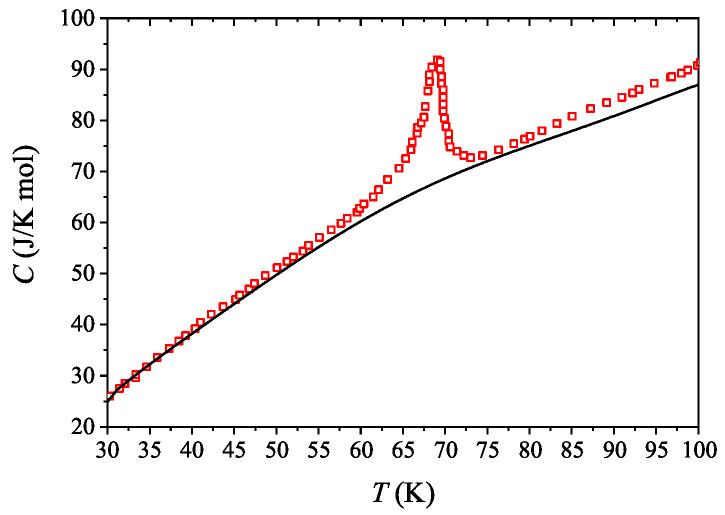
Red squares are the experimental data presented in Reference [6], black curve is the fitted specific heat with the additional peak at $T_{\mathrm{MI}}$ subtracted.

The specific heat curve, measured in Reference [6], is presented without the scattering effect, for example, without the additional peak in Figure 2 in the temperature range of 30–100 K. This fitted curve involves the specific heat related to the phonons below the transition temperature of 69 K. Above the transition temperature it consists of the specific heat contribution of phonons and conducting electrons.

The energy contribution of the electron scattering is approximated by a Lorentzian energy distribution

(1) $$g\left(\varepsilon \right)=\frac{\Gamma /2}{{\left(\varepsilon_{r}-\varepsilon \right)}^{2}+{\left(\Gamma /2\right)}^{2}},$$

where Γ is the half width of the distribution, which is related to the scattering rate or the inverse momentum lifetime, $\varepsilon_{r}$ is the temperature dependent resonant energy—this is the maximum value that an electron can accumulate during the scattering event and it has a maximum value at T=69 K, and ε is the instant electron energy [20]. It is assumed that the resonance has a maximum at the metal-insulator (structural) transition, for example, where $T_{\mathrm{res}}=T_{\mathrm{MI}}$. From the width of the specific heat peak it can be physically assumed that the electrons can contribute to the effect from a wider temperature range around the resonant temperature. Thus, a relevant Gaussian distribution function for the resonant energy

(2) $$\varepsilon_{r}=\varepsilon_{0}\;\exp\left(-\varepsilon_{1}{\left(T-T_{\mathrm{res}}\right)}^{2}\right)$$

can adequately express the physical situation. The parameter $\varepsilon_{1}$ controls the width of the peak and expresses that the scattering effect is going below smaller and above the higher temperature than the transition temperature.Thus, the scattering distribution is a function of temperature as well g(ε,T).

**Figure 3:**
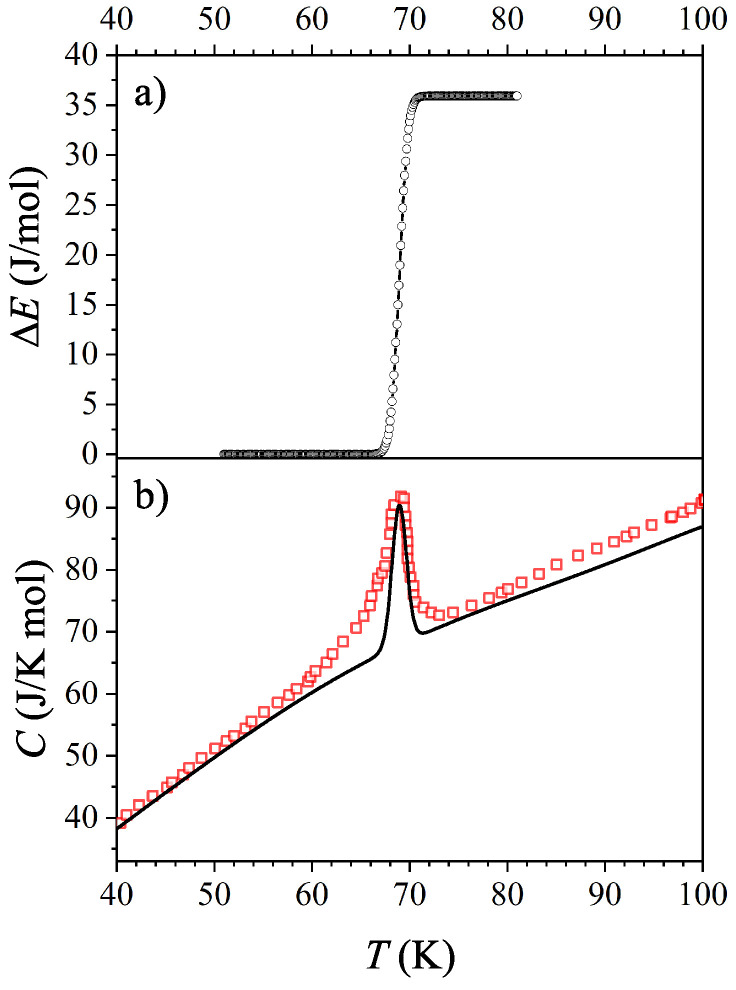
(**a**) The temperature dependence of energy increase due to internal electron scattering. (**b**) Calculated specific heat of BaVS${}_{3}$. Please note that the baseline, which contains the phononic (and electronic for the metallic regime) contribution, is fitted to the experimental data and only the additional peak caused by the enhanced scattering process is calculated.

The calculation of the energy increase due to the scattering starts from a reference temperature $T_{\mathrm{ref}}$ to the maximal temperature $T_{\max}$, presently in our calculations 30 K and 100 K. The difference is denoted by $T_{\mathrm{up}}=T_{\max}-T_{\mathrm{ref}}$. The generated energy increase can be calculated by temperature steps *N*, taking into account the continuous change of temperature. Here, we calculate the energy change between the $T_{i}-T_{i+1}$ as

(3) $$\Delta \varepsilon_{i}\left(T_{i}\right)=\frac{N_{0}}{N}\int_{0}^{\infty }\varepsilon \;g\left(\varepsilon ,T_{i}\right)f\left(\varepsilon ,T_{i}\right)\;d\varepsilon ,$$

here *f* is the Fermi-Dirac distribution; furthermore,

(4) $$T_{i}=T_{\mathrm{ref}}+\frac{i}{N}T_{\mathrm{up}}$$

is the instantaneous temperature between the range $T_{\mathrm{ref}}<T_{i}<T_{\max}$ using the notion i=0,1,2,⋯,N. The total energy change is therefore the sum of the $\Delta \varepsilon_{i}\left(T_{i}\right)$ terms as

(5) $$\Delta E\left(T\right)=\sum_{i=1}^{n}\Delta \varepsilon_{i}\left(T_{i}\right),$$

where $T=T_{\mathrm{ref}}+\frac{n}{N}T_{\mathrm{up}}$ with n∈[1,N]. The resulting energy increase is plotted in Figure 3a, from where it can be seen that the internal energy has a stepwise increase at the metal-insulator transition. The specific heat is the temperature derivative of the internal energy

(6) $$C={\left[\frac{1}{\tilde{n}}\frac{\partial E}{\partial T}\right]}_{V},$$

where $\tilde{n}$ is the amount of substance. Adding the phononic (and electronic in the case of the metallic regime) contribution to the specific heat from Figure 2 to the derivative of energy increase in Figure 3a we obtain a peak at 69 K, as presented in Figure 3b. The obtained curve agrees well with the experimental results.

The very same calculations can be done for the sample prepared by Imai et al. [2]. The calculated parameters are collected in Table 1 for the two samples.

Here we wish to emphasize that Γ and $\varepsilon_{1}$ are strongly dependent on sample purity, as defects and sample purity clearly cause a higher momentum relaxation rate, which in extreme cases can hinder the observed enhanced electron scattering. In principle these two parameters can also characterize the further samples in terms of purity.

## 4. The Heat Conductivity Peak

An interesting consequence of the scattering effect is a small peak in the heat conductivity at 69 K as seen in Figure 4 in Reference [6] (Figure A2 of Appendix A). In metals the total heat current due to the electrons [20] can be written as

(7) $$J_{q}=-\frac{1}{3}n_{e}vl\frac{\partial u}{\partial z},$$

where $n_{e}$ is the electron density, *v* is the electron speed, *l* is the mean free path, *u* is the transported internal energy by one electron and *z* is the spatial coordinate. Since,

(8) $$\frac{\partial u}{\partial z}=\underset{c}{\underset{︸}{\frac{\partial u}{\partial T}}}\frac{\partial T}{\partial z},$$

where *c* is the heat capacity per electrons and introducing $C=n_{e}c$, it is possible to connect heat capacity with heat conductivity by the relation

(9) $$\kappa =\frac{1}{3}Cvl.$$

In order to explain the heat conductivity peak caused by the heat capacity peak first we need to estimate of the electron speed in the scattering process. We take an off-resonant value at 66 K from the graphs in Reference [6] (Figure A2 of the Appendix A), where $\kappa_{1}=0.82$ W/K·m and $C_{1}=80$ J/K mol $=6.6\times 10^{5}$ J/ K m${}^{3}$. It is supposed that $v_{1}=0$ m/s, since the material is in the insulator phase where the conduction of heat is caused by the phonons only. In the second step, values at resonance T=69 K are taken: $\kappa_{2}=0.84$ W/ K·m and $C_{2}=92$ J/K mol $=7.6\times 10^{5}$ J/K m${}^{3}$. The BaVS${}_{3}$ is known to be a bad metal where the mean free path of electrons is in the order of V−V distance, typically l≈0.28 nm. In the metallic state, the electrons also provide a contribution to the heat conduction. Without this, the heat conductivity should decrease as it can be read out from the tendency of the curve. The total change of heat conductivity due to the resonant electron scattering can be approximated as $2(\kappa_{2}-\kappa_{1})$. Taking into account Equation (9), the following relation can be obtained:

(10) $$2\left(\kappa_{2}-\kappa_{1}\right)=\frac{1}{3}C_{2}v_{2}l.$$

The calculated electron speed is $v_{2}=560$ m/s, which is large enough to prove that the heat capacity peak is directly related to the peak in heat conductivity and also caused by the enhanced (resonant) electron scattering.

Please note that the peak in the heat conductivity has a local maximum instead of a local minimum. From a, for example, normal electron-electron scattering one might expect an inverse behavior; however, here, due to a structural reorientation there is an effective drag force which acts on the electrons. These undergo the resonant scattering event and therefore, due to a collective motion, a local maximum is also possible.

## 5. Conclusions

Several phenomena may play a role in the extra heat capacity of BaVS${}_{3}$ at the temperature 69 K of a metal-insulator transition. Yet, the contribution of these effects is not enough to explain the rather observed peak. The present work shows that an internal electron scattering process, due to the structural transition, may carry such addition energy that leads to a strong increase of heat capacity around the transition point. The peak in the heat conductivity is also explained.

## Figures and Tables

**Table 1:** List of calculated parameters for the two different samples.

Variables	Sample from Reference [6]	Sample from Reference [2]
Γ	$6\times 10^{-23}$ eV	$60\times 10^{-23}$ eV
$\varepsilon_{0}$	6meV	6meV
$\varepsilon_{1}$	$0.5\;{1/K}^{2}$	$0.08\;{1/K}^{2}$
